# Photoreceptor damage induced by low-intensity light: model of retinal degeneration in mammals

**Published:** 2013-07-25

**Authors:** Maria Ana Contín, Milagros M. Arietti, María M. Benedetto, Claudio Bussi, Mario E. Guido

**Affiliations:** Centro de Investigaciones en Química Biológica, (CIQUIBIC, UNC–CONICET), Departamento de Química Biológica, Facultad de Ciencias Químicas,UNC, Haya de la Torre y Medina Allende, Ciudad Universitaria, X5000HUA, Córdoba, Argentina

## Abstract

**Purpose:**

Retinal degeneration caused by a defect in the phototransduction cascade leads to the apoptosis of photoreceptor cells, although the precise molecular mechanism is still unknown. In addition, constant low light exposure produces photoreceptor cell death through the activation of downstream phototransduction. The authors investigated the time course and molecular mechanisms of death and the rhodopsin phosphorylation occurring during retinal degeneration after exposure to continuous low-intensity light.

**Methods:**

Wistar rats were exposed to constant cool white 200 lx intensity LED light (LL) for one to ten days and compared with animals kept in the dark (DD) or controls exposed to a regular 12:12 h (LD) cycle. One eye from each rat was used for histological and quantitative outer nuclear layer (ONL) analysis and the other for biochemical assays.

**Results:**

The histological analysis showed a significant reduction in the ONL of LL-exposed rats after seven days compared with LD- or DD-exposed rats. Retinal analysis by flow cytometer and the TUNEL assay revealed an increase in cell death in the ONL, the in vitro enzymatic activity assay and western blot analysis showing no caspase-3 activation. The rhodopsin analysis demonstrated more phosphorylation in serine 334 residues (Ser^334^) in LL-exposed than in LD- or DD-exposed rats. However, for all times studied, rhodopsin was completely dephosphorylated after four days of DD treatment.

**Conclusions:**

Constant light exposure for seven days produces ONL reduction by photoreceptor cell death through a capase-3-independent mechanism. Increases in rhodopsin-phospho-Ser^334^ levels were observed, supporting the notion that changes in the regulation of the phototransduction cascade occur during retinal degeneration.

## Introduction

Retinal degeneration (RD) caused by defects in the phototransduction mechanism is generally characterized by photoreceptor cell death as a result of genetic mutations, vitamin A deficiency, or prolonged light exposure [1-4]. Although the functional alteration and disease mechanisms involved in RD may differ depending on the gene affected, the common result is cell death by apoptosis [1,5-9]. Retinal damage by light exposure, leading to cell death in the visual cortex via a series of apoptotic events, has served as a model for human RD arising from environmental insult, aging and genetic diseases [10]. The phenomenon of retinal light damage is a visual pigment-mediated process [11] associated with both long exposure times and shorter wavelength light exposure. The exposure of retinal tissue to radiant energy can generate free radicals, with the retina being unable to overcome the protective mechanism to revert this process (reviewed in [12]). In 1966, Noell et al. [13] suggested that low-intensity light can also cause damage to the retina, and there is evidence that rod photoreceptors exposed to low-intensity light die from a light-induced constitutive signal transduction mechanism [14,15].

Apoptosis is manifested by the appearance of double-stranded DNA breaks within the initial hours of light exposure, which depends on the wavelength and intensity of light used [2,7,16,17]. However, there are contradictory results regarding the apoptotic mechanism and the role of caspase-3 in light-induced models: some authors have attributed a central role to this enzyme in photoreceptor degeneration [18-20], whereas others have reported a caspase-3-independent mechanism associated with the role of Ca^+2^-dependent protease calpains or cathepsin D as alternative death pathways [20-24]. It is clear from all these findings that photoreceptor death varies in both severity and its apoptotic mechanisms, which depend on the strain, light intensity and wavelength used.

Hao et al. [25] provided evidence of two apoptotic pathways that are initiated by light activation of rhodopsin. Bright light triggers apoptosis of photoreceptor cells through a mechanism requiring activation of rhodopsin but not of the phototransduction mechanism, whereas low light intensities induce photoreceptor apoptosis by photopigment activation and subsequent downstream signal transduction [25]. In albino rats, retinal stimulation with continuous low white light causes the progressive deterioration of photoreceptors, an effect that does not occur in rats exposed to cyclic illumination conditions [13,26-31]; the threshold cyclic light intensity that produces damage to the retinas of albino rats lies around 270 lx [32]. Although light microscope findings revealed fragmentation and disorientation of the photoreceptor outer segments (OS) after three to five days of constant exposure to low light, with no photoreceptors at all remaining after thirty days of exposure [33], no single change could be identified that would inexorably lead to cell death [33]. Ultrastructural changes in photoreceptors suggest that death occurs because the cells can no longer maintain their anabolic processes [34-36], but elucidation of the precise mechanisms involved calls for the systematic study of photoreceptor cell functions.

We consider that a better understanding of the molecular mechanisms triggered by low light intensity exposure leading to RD could provide valuable insights into the progression of clinical disorders related to phototransduction defects. The aim of this work, therefore, was to investigate the time course of RD and the mechanisms of death in Wistar rats stimulated by constant exposure to 200 lx of diffuse, cool white, LED light. Our results show that continuous illumination produces retinal damage, with a reduction in the ONL occurring after seven days of light stimulation through a caspase-3-independent mechanism. Moreover, rhodopsin protein expression analysis showed that rhodopsin levels did not reduce before ONL cells died, but became more phosphorylated at Ser^334^ residues.

## Methods

### Animals

All animal procedures were performed in accordance with the ARVO statement for the use of animals in ophthalmic and vision research, which was approved by the local animal committee (School of Chemistry, UNC, Exp. 2013–291). Male adult Wistar rats aged 12–15 weeks were exposed to a 12:12 h light dark (LD) cycle, with the light on (less than 10 lx) from zeitgeber time (ZT) 0 to 12 for all animals from the time they were born up to the experiment. Food and water were available ad libitum.

### Light damage

Retinal degeneration was induced in a temperature-controlled light stress box equipped with 200 lx of diffuse, cool white, LED lamps fixed on the inner upper surface, which illuminated the interior white walls. The illumination at the level of the rats’ eyes was measured as 200 lx with a light meter (model 401036; Extech Instruments Corp., Waltham, MA). All rats were killed in a CO_2_ chamber at ZT 12 at one to ten days after constant light stimulation (LL) according to the experimental design.

Control experiments were performed by exposing all animals to 200 lx under a 12:12 h LD cycle, with light on at ZT 0 and off at ZT 12. To verify that the LD cycle did not induce degenerative damage during the light stimuli as observed in constant light (LL), other animals were subjected to constant darkness (DD) during the same times as animals exposed to the LD cycle and under identical conditions in the temperature-controlled stress box.

### Histological and quantitative outer nuclear layer analysis

The methods employed for fixation, embedment, sectioning and histological analysis of eyes were as previously described [37]. Briefly, rat eyes at different days after light or control treatment were fixed in 4% (W/V) paraformaldehyde in 100 mM sodium phosphate buffer (PBS, pH 7.3) overnight at 4 °C, before being cryoprotected in sucrose and mounted in an optimal counting temperature compound (OCT; Tissue-Tek® Sakura). Retinal sections 10 µm thick were then cut along the horizontal meridian (nasal-temporal) using the cryostat. The sections were lightly stained with 1% (W/V) propidium iodide (PI) for two minutes and photographed using a confocal microscope (Olympus FV300, Japan) at 40× magnification. The number of ONL nuclei was counted on the photograph of each of the four designated retinal areas – left, middle left, middle right and right – for ten different animals per treatment. Quantitative ONL analysis was performed to quantify photoreceptor survival on the entire ONL (width and length of the micrograph), using the software ImageJ (v. 1.45), and the plugin “Automatic Nuclei Counter” [38].

### FACScan flow cytometer

Retinas were dissected, dissociated in 0.25% (W/V) trypsin (Life technology, Inc.) and processed as described previously [20] with modifications. Briefly, cells were washed twice and then resuspended with cold PBS (100 mM sodium phosphate). Aliquots of 100 μl were separated and loaded into a Neubauer chamber for counting. After labeling 1×10^4^ cells by adding 5 μl of FITC Annexin V (PE Annexin V Detection Kit I, 559763, BD Biosciences) and 10 μl of propidium iodide (PI 0.05 mg/ml stock solution, Sigma, P48864), the solution was gently vortexed, incubated for 15 min at room temperature (25 °C) in the dark and analyzed by flow cytometry on a Becton-Dickinson FACS flow cytometer within the first hour. The cell data of six retinas per treatment from three independent experiments were analyzed by the FlowJo software.

### TUNEL staining

The terminal deoxynucleotidyl transferase dUTP nick end labeling (TUNEL) assay was performed following the procedures provided with the kit from Roche Diagnostics Corp. (Indianapolis). Briefly, frozen sections of rat retinas were cut on a cryostat before being postfixed with 4% paraformaldehyde and permeabilized in PBS, 0.1% (W/V) sodium citrate, 0.1% (V/V) Triton X-100. The reaction mixture (50 μl TUNEL) was added to each sample and the slides were incubated in a humidified atmosphere for 60 min at 37 °C in the dark. Then, sections were lightly stained with 1% (W/V) propidium iodide (PI) for two minutes. For negative controls, the TdT enzyme was replaced by label solution, and the samples from three different animals per treatment were analyzed by a confocal microscope (Olympus FV300, Tokyo, Japan).

### Outer segment preparation

Animals were killed in the dark under infrared illumination. The eyes were washed once with PBS buffer and the retinas removed and maintained in 20% (W/V) sucrose in the dark. The OS were purified by sucrose gradient purification as described previously with some modifications [39]. Briefly, the retinas were put on ice in a glass tube containing an isolation medium composed of 20% sucrose PBS in the presence of protease inhibitor cocktail P33360 (Invitrogen) and phosphatase inhibitor cocktail tablets (PhosphoSTOP, Roche), and were homogenized with five up and down passes in a Dounce homogenizer. The retinal homogenate (500 µl) was layered on top of a discontinuous sucrose gradient at concentrations of 20%, 27%, and 50% (W/V) sucrose, before being centrifuged at 141,000 ×*g* for 2 h at 4 °C in a Beckman ultracentrifuge, 50.2 TI rotor (WS-BECKOPTXL90). After ultracentrifugation, the rod OS sedimenting between the 27 and 50% sucrose phase were collected, diluted in PBS, and centrifuged at 7700 ×*g* for 10 min at 4 °C before being suspended in PBS buffer. The sediment collected from the ultracentrifugation at 141,000 ×*g* in the sucrose gradient was resuspended in PBS and centrifuged at 800 ×*g* for 10 min, with the procedure repeated three times. This preparation was named pellet preparation (P).

### Western blot

#### Caspase-3

Homogenates of whole rat and embryonic chick retina (embryonic stage 11, E11) resuspended in 200 µl PBS buffer containing the protease inhibitor cocktail were lysed by repeated cycles of ultra-sonication and the total protein content was determined by the Bradford method [40]. Chick retina served as a positive control of caspase-3 expression [41]. Then, the homogenates of whole rat and embryonic chick retinas were resuspended in sample buffer (SB: 62.5 mM Tris HCl pH 6.8; 2% (W/V) SDS; 10% (V/V) glycerol; 50 mM DTT; 0.1% (W/V) bromophenol blue) and heated at 90 °C for 5 min. The proteins (20 µg) were separated by SDS-gel electrophoresis on 12% polyacrylamide gels, transferred onto nitrocellulose membranes, blocked for 1 h at room temperature with 5% (W/V) skim milk in PBS and then incubated overnight at 4 °C with antibody against caspase-3 (HPA002643; Sigma, St Louis, MO) in the incubation buffer (2.5% (W/V) skim milk and 0.1% (V/V) Tween-20 detergent in PBS). Membranes were washed three times (15 min each wash) in washing buffer (PBS containing 0.1% Tween-20; Sigma, P1379), and then incubated with the corresponding secondary antibody (goat anti-rabbit IRDye Odyssey LI-COR) in the incubation buffer for 1 h at room temperature, followed by three washes (15 min each wash) with washing buffer. Membranes were scanned using an Odyssey IR Imager (LI-COR Biosciences) before being stripped with 0.5 M NaOH and incubated for 1 h at room temperature with α-tubulin (Sigma T6199) antibody. They were then washed three times (15 min each wash) with washing buffer and incubated with secondary antibody (goat anti-mouse IRDye ® 800CW) in the incubation buffer for 1 h at room temperature before carrying out three further washes of 15 min each with washing buffer. Membranes were scanned using an Odyssey IR Imager and two retinas from four independent experiments were analyzed for each time point.

#### Rhodopsin

Homogenates of whole rat retinas, resuspended in 200 µl PBS buffer containing the protease inhibitor cocktail, were lysed by repeated cycles of ultra-sonication. As the degenerated retinas (LL) contained less protein than control retinas (LD), and as we wished to compare the ratio of rhodopsin in LL to that in LD, rather than the absolute protein quantity, the same amount of samples per retina (not total protein) were loaded. Homogenates (200 µl) were resuspended in sample buffer and 40 µl were separated by SDS-gel electrophoresis on 15% polyacrylamide gels. The gel was then transferred onto nitrocellulose membranes, blocked for 1 h at room temperature with 5% skim milk in PBS and then incubated overnight at 4 °C with rhodopsin antibodies (Ret-P1 Sigma) in the incubation buffer. The membranes were subsequently washed, incubated with the secondary antibody goat anti-mouse IRDye 800CW and scanned in the Odyssey IR Imager as indicated in the caspase-3 western assay. Six retinas from four independent experiments were analyzed for each time point. Densitometry of western blots was performed using the ImageJ software and the ratio from the quantitative analysis of three oligomer band optical densities in LL relative to LD was expressed as a percentage of change.

#### Rhodopsin phosphorylation

The total proteins of the OS and P preparation were quantified by the Bradford method [40] and 40 µg were separated by SDS-gel electrophoresis on 15% polyacrylamide gels. The gel was then transferred onto nitrocellulose membranes, blocked and incubated overnight at 4 °C with rhodopsin phosphorylated antibodies (Phospho-Ser334 assay Biotech) and secondary antibody goat anti-mouse IRDye ® 800CW, and scanned in the Odyssey IR Imager as indicated in the caspase-3 western assay. The membranes were subsequently stripped with 0.5 M NaOH and incubated overnight at 4 °C with α-tubulin (Sigma T6199) antibody and scanned using an Odyssey IR Imager. Four retinas from three independent experiments were analyzed for each time point. Densitometry of western blots was performed using the ImageJ software and the ratio from the quantitative analysis of three oligomer band densities of rhodopsin to tubulin in OS samples was expressed as the relative optical density.

### Caspase-3 activity

Retinal caspase-3 activity was measured with a commercial colorimetric kit (EnzChek Caspase 3 Assay Kit # 1; Molecular Probes Inc. Eugene, OR) according to the manufacturer’s instructions. Briefly, equal aliquots of retinal homogenate were incubated at 37 °C for 30 min with caspase-3-specific substrate Z-DEVD-AMC in the kit reaction buffer. The absorbance of each sample was read at 441 nm and caspase-3 activity levels were directly proportional to the color reaction. Chick retinas (embryonic stage 11) were used as positive control of caspase-3 activity. Results of each LL treatment were expressed as the percentage change in the absorbance measured in LL with respect to LD.

### Statistical analysis

Data are expressed as mean ± standard derivation (SD). Statistical comparisons were made using a one-way ANOVA (ANOVA); p value <0.05 was considered statistically significant. To prove the normality and homogeneity of variance assumption, we used Shapiro-Wilks and Levene tests. In all cases with significance, a Duncan test was applied. A p value <0.05 was considered statistically significant. When we worked with percentage data we applied a logarithm transformation and when data did not present normality, a non-parametric Kruskal-Wallis test was conducted.

## Results

### Histological and quantitative outer nuclear layer analyses

**Figure 1:**
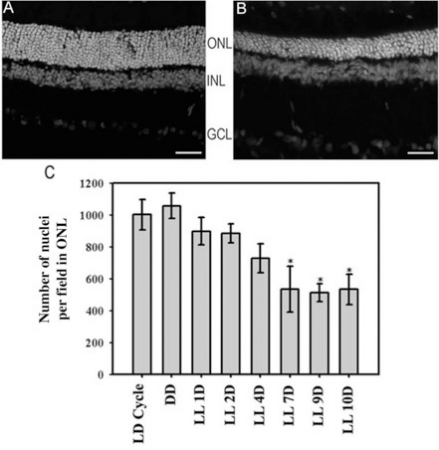
Effect of constant low light exposure on the number of outer nuclear layer nuclei. **A:** Retinas reared in a 12:12 h light (200 lx) dark cycle (LD Cycle). Photoreceptors in ONL were morphologically normal. **B:** Retinas from rats exposed to ten days of constant 200 lx light (LL). These ONL became thinner than the ONL in A. ONL, outer nuclear layer; INL, inner nuclear layer; GCL, ganglion cell layer. Scale bar indicates 30 µm. **C:** Numerical quantification of ONL nuclei. Rat retina images corresponding to the LD cycle, non-exposure to light (DD) or exposure to 200 lx light (LL) for 1,2,4,7, 9 and 10 days were analyzed to quantify photoreceptor survival. The number of nuclei in the different areas (left, middle left, middle right and right) of the ONL was counted using the Automatic Nuclei Counter plug-in (v1.45) for ImageJ software with 40×magnification. Data are mean ± SD (n=2 animals/group) from five independent experiments, p=0.025, by a one-way ANOVA; *:p<0.05 by Duncan’s post- hoc test (LL 7, 9 and 10 D versus control in the LD cycle).

As described in the Methods section, retinas of animals exposed to cool white 200 lx light (LL) were processed by quantitative ONL analysis to assess retinal layer thinning. Photoreceptor damage occurred in the light-exposed eyes and resulted in the loss of around 50% of the ONL photoreceptor nuclei at ten days of LL stimulation (Figure 1B) compared with animals reared in an LD cycle (Figure 1A and Appendix 1). There was a significantly lower number of nuclei in the ONL of LL-exposed groups after seven days of light treatment (535.25 nuclei ± 142.99; Figure 1C) compared with the LD Cycle (1003.22 nuclei ± 95.32) or DD (1058.23 nuclei ± 79.31).

### Analysis of death

#### FACScan flow cytometer

Biochemically, apoptosis is characterized by the phospholipid phosphatidylserine (PS) becoming enriched in the outer leaflet of the plasma membrane. Annexin V is a 35–36 kDa Ca^2+^-dependent phospholipid-binding protein that has a high affinity for PS [42,43] and is used to differentiate apoptotic from necrotic cells [42]. Early on in apoptotic cells, membrane PS is translocated from the inner to the outer leaflet of the plasma membrane. When apoptosis is measured over time, early Annexin V labeling is positive and PI negative, with the membrane still retaining its integrity. However, when both Annexin V and PI are positive, this indicates that the membrane has lost its integrity and signals the end stage of apoptosis and death.

**Figure 2:**
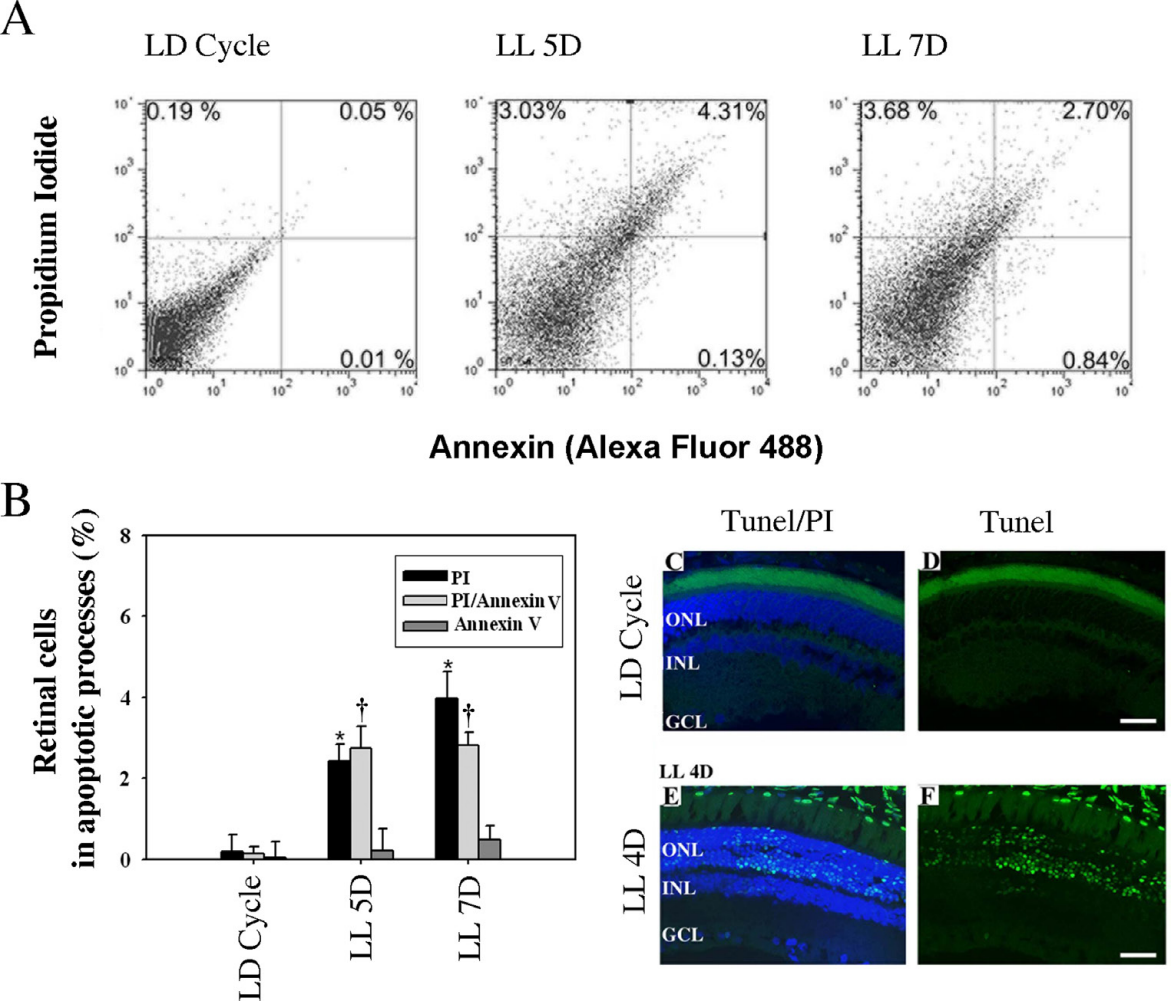
Annexin V, PI and TUNEL assay in rat retina exposed to low-intensity light. **A:** Image showing a representative analysis of retina from rats exposed to a regular LD cycle 200 lx (L12h D12h) and for five and seven days of constant light (LL). **B:** Quantitative analysis of flow cytometric detection, showing the mean of three independent experiments of retinal cells undergoing cell death labeled with PI (quadrant 1), PI plus Annexin V (quadrant 2) and Annexin V (quadrant 4). Data are mean ± SD (n=3 animals/group) from three independent experiments. PI: p=0.012, by a one-way ANOVA. *: p<0.05 by Duncan’s post-hoc test (LL5D and LL7D versus control in the LD Cycle). PI/Annexin: p=0.0153, by a one-way ANOVA. †: p<0.05 by Duncan’s post- hoc test (LL5 and LL 7D versus control in the LD cycle). Annexin: p=0.1455, by a one-way ANOVA. **C-F**: TUNEL staining (green) showing negative labeling in rat retina reared in LD cycle **(C,D)** and nucleosomal DNA fragmentation in retinas exposed to four days of constant light LL 4D **(E,F)**. The images are representative of 3 different experiments per treatment. Blue: Propidium iodide total DNA labeling **(C,E).** Scale bar indicates 30 µm.

Figure 2A,B presents the temporal quantification of Annexin V and PI staining by flow cytometer studies. Figure 2B shows that after five days of LL exposure, PI and Annexin V increased in retinal cells (LL 5D, PI:2.43±0.41%; PI/Annexin V:2.75±0.54% and Annexin V:0.22±0.17%) compared with control cells (LD Cycle: PI:0.19±0.42%; PI/Annexin 0.13±0.14%, Annexin V 0.04±0.03%). At seven days, PI increased and PI/Annexin decreased with respect to LL 5D (LL 7D: PI:3.98±0.64%; PI/Annexin: 2.81±0.33% and Annexin V:0.49±0.33%). These results indicate that exposure of Wistar rats to LL produces retinal cell death.

To demonstrate that the cell deaths occurred in the ONL where the photoreceptor nuclei reside, we analyzed DNA fragmentation using the TUNEL assay in the retinal histological section. These results showed positive TUNEL staining (green) localized at the ONL in the retinas of rats exposed to four days of LL (Figure 2 E,F), whereas no staining was detected after four days in retinas in the LD Cycle (Figure 2 C,D).

#### Caspase-3 analysis

**Figure 3:**
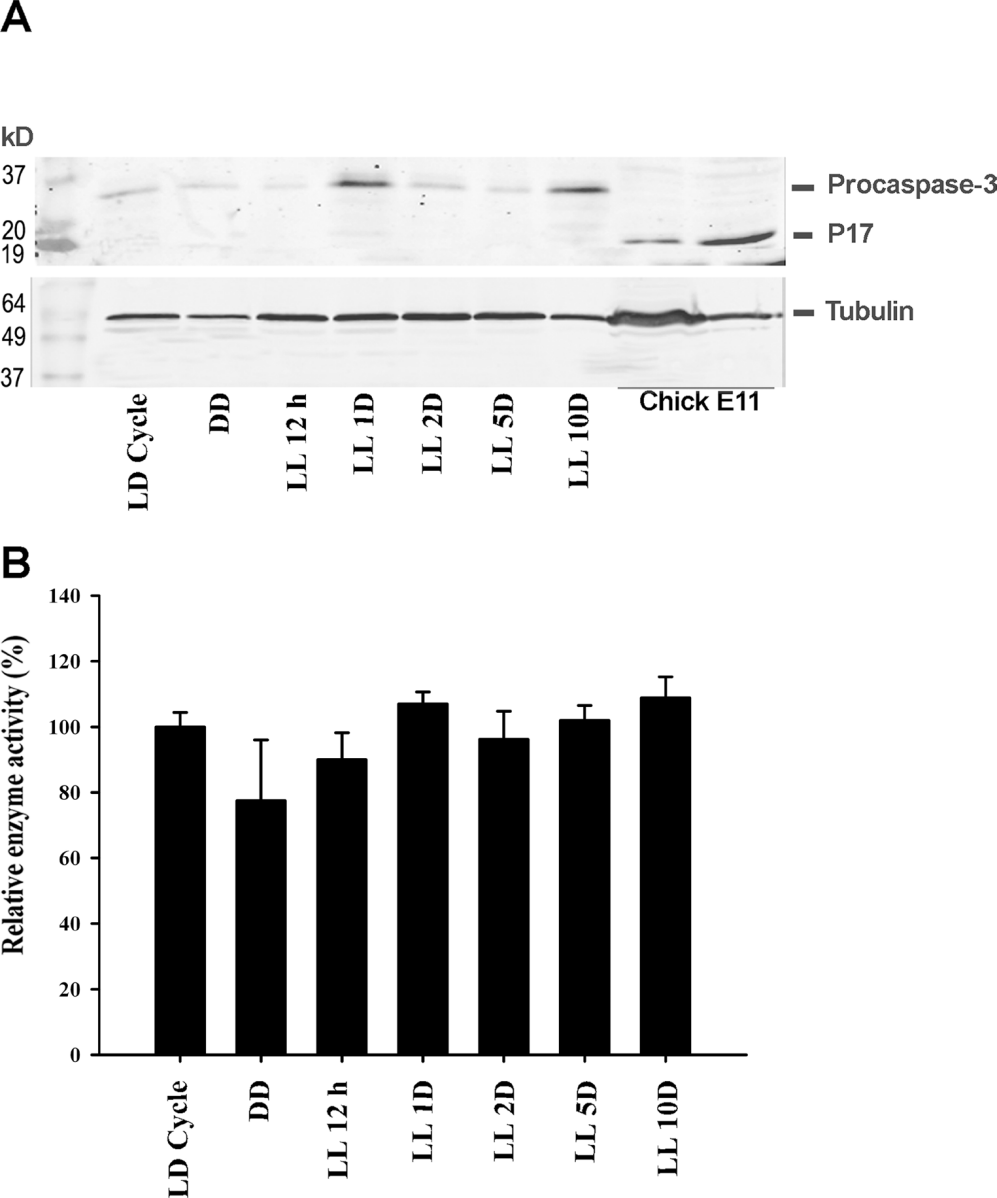
Caspase-3 analyses of rat retinas exposed to low-intensity light. **A**. A western blot showing caspase-3 in retinas after exposure to a regular LD cycle of 200 lx (LD Cycle; 12h L:12h D) or to 12 h or to one, two, five or ten days of constant light (LL), respectively. A positive control from embryonic chick retina is also included. Results are representative of five independent experiments. **B**: Caspase-3 activity by detection of DEVD-pNA cleavage was measured in rat retinas at the same time as that in A. The results are expressed as the enzyme activity in LL relative to that in retinas maintained in LD (%). Data are mean ± SD (n=2 animals/group) from four independent experiments. Non-parametric Kruskal-Wallis test showed no significant statistical differences (p=0.7631). The results are expressed as percentages.

The expression of caspase-3 protein and its activity was investigated during photoreceptor cell death using two different analytical techniques (Figure 3). The immunoblot analysis of retinas from animals exposed to LL stimulation revealed pro-caspase-3 protein (32 kDa) to be present at all times studied, although no active form of the protein (17 kDa) was observed under any condition tested (Figure 3A). However, a fraction of cleaved caspase-3 immunoreactivity was detected in the homogenates of control tissue (chicken retina at embryonic stage 11).

Caspase-3 activity was further assessed by measuring the cleavage of the colorimetric substrate DEVD-pNA (Figure 3B). The results in Figure 3B show the average absorbance measured in each LL and DD treatment compared with LD controls (%). In agreement with the results of the immunoblot analysis, no significant activation of caspase-3 was observed in the retinas at any of the examined times compared with the control group (chicken embryonic retinas; data not shown), strongly suggesting that the cell death mechanism in photoreceptors was caspase-3-independent.

### Rhodopsin analysis

**Figure 4:**
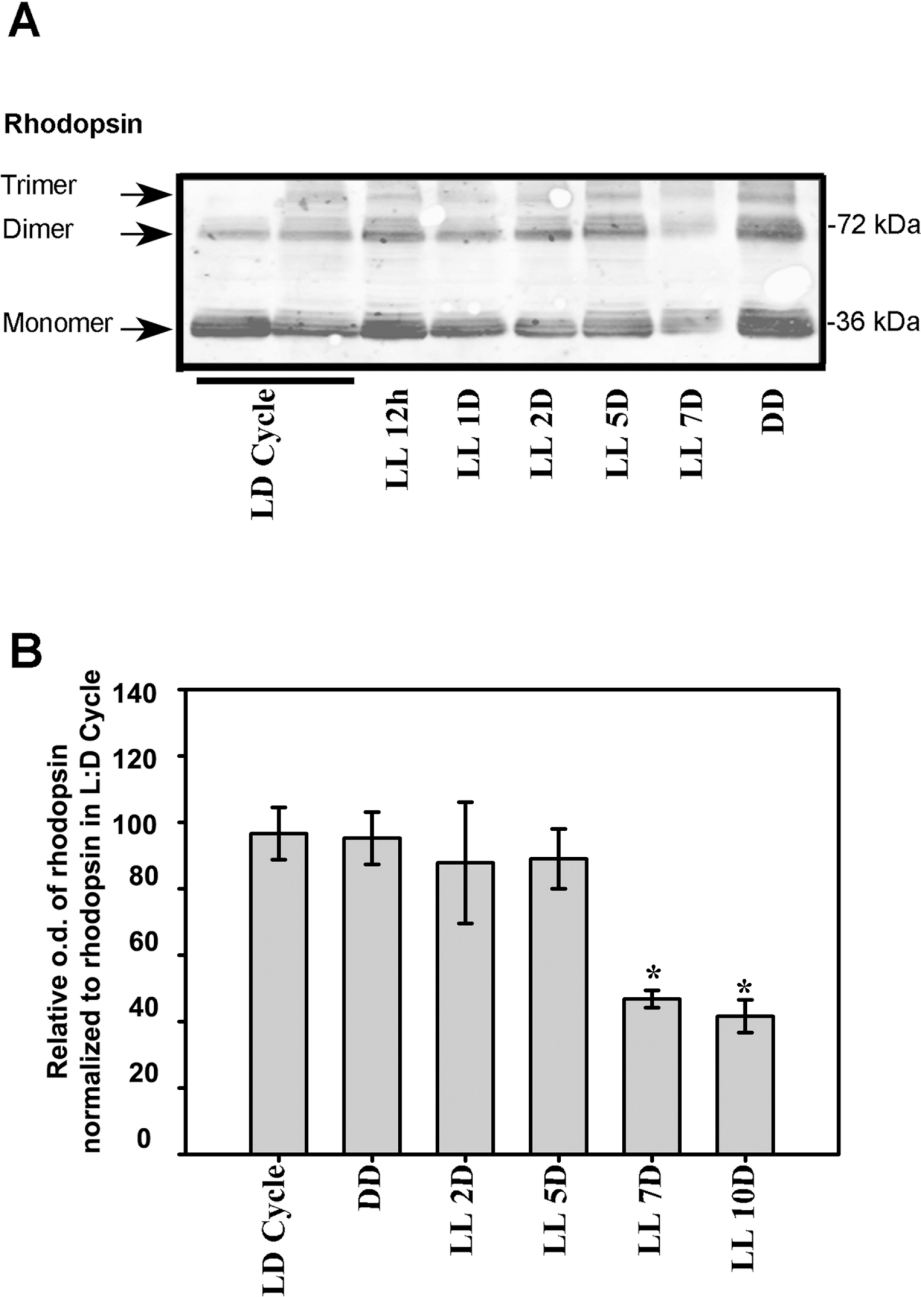
Analysis of rhodopsin expression in rat retina exposed to low-intensity light. **A**: A western blot immunolabeled rhodopsin with a specific antibody showing the predicted isoform bands. The margin shows the oligomerization states of the rhodopsin fragment (left) and the molecular mass (right). **B:** Quantitative analysis of the three oligomer band optical densities (o.d.) normalized to the content of rhodopsin from the LD cycle controls. Data are mean ± SD (n=3 animals/group) from four independent experiments. Transformed data to log_10_ showed p=0.0001 by a one way-ANOVA. Duncan’s post-hoc test *: p<0.05, (LL7 and LL 10 D versus control in the LD cycle. The results are expressed as percentages.

**Figure 5:**
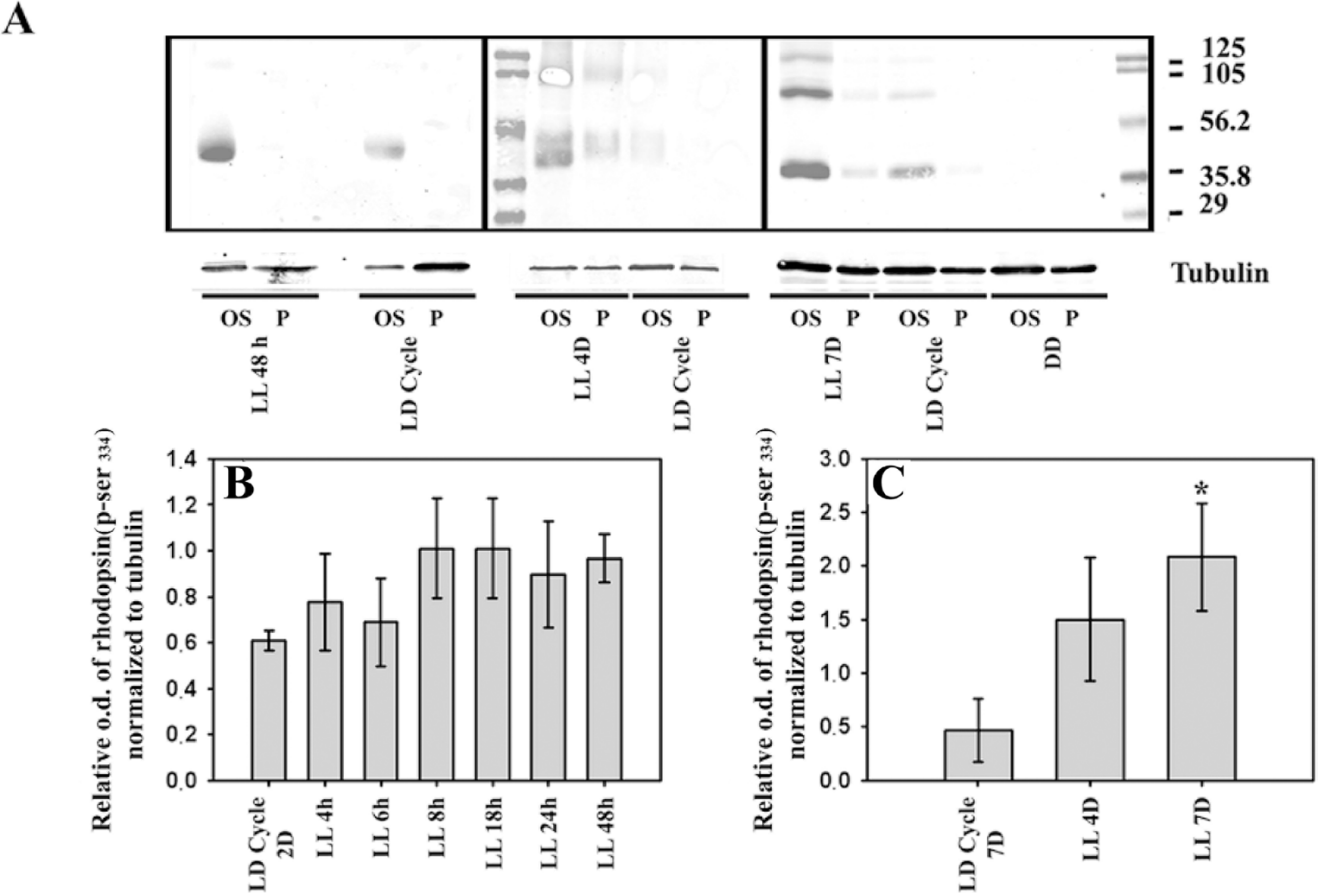
Analysis of rhodopsin phosphorylated in Ser^334^ in the outer segment (OS) and pellet (P) of rat retinas exposed to low-intensity light. **A**: A western blot of rhodopsin (phospho-Ser^334^) in retinas after exposure to a regular light dark cycle (LD Cycle; 12 h L 200 lx: 12 h D) or to 48 h, 4 or 7 days (LL 48 h, LL4D and LL 7D) of constant light. Experimental animals kept in the dark (DD) are also included. The margin shows the molecular mass (right). **B**: Rhodopsin (phospho-Ser^334^) quantification of OS fraction in retinas after exposure to LD Cycle 48 h (12 h L 200 lx:12 h D), 4, 6, 8, 18, 24 or 48 h of constant low light (LL 4h, LL 6 h, LL 8 h, LL 18 h, LL 24 h and LL 48 h). Data are means ± SD (n=2 animals/group) from three independent experiments. One-way ANOVA showed no significant statistical differences (p=0.170). **C:** Rhodopsin (phospho-Ser^334^) quantification of OS fraction in retinas after exposure to LD Cycle of seven days (12 h L 200 lx:12 h D) or four or seven days of constant low light (LL 4D, LL 7D). Data are means ± SD (n=2 animals/group) from three independent experiments, p=0.015 by a one-way ANOVA; *: p<0.05 by Duncan’s post- hoc test (LL 7D versus control in the LD cycle).

Rhodopsin expression and phosphorylation, known to be involved in the critical quenching step of the phototransduction cascade, were evaluated to investigate whether the photopigment of the phototransduction cascade was affected by low light stimulation within the photoreceptor cells. As shown in Figure 4, western blot of retinas immunolabeled with rhodopsin antibody showed the predicted isoforms of this protein [44]. After seven days of LL stimulation, protein levels were lower than those in LD controls and DD, with the highest levels of rhodopsin immunoreactivity being recorded in the latter (Figure 4A). Densitometric analysis of the rhodopsin levels showed a significant reduction after seven days of LL, coinciding with a reduction in ONL (Figure 4B) and suggesting that constant low light did not affect rhodopsin expression before apoptotic photoreceptor cell death. The effects of low light stimulation stress on rhodopsin phosphorylation (Ser^334^ residue) was studied next in the OS and P preparation by western blot methods involving a specific antibody against rhodopsin-phospho-Ser^334^, as described in the Methods section. In Figure 5A, a representative western blot of the positive immunoreactivity of rhodopsin Ser^334^, is shown at two, four and seven days of LL exposure. As can be seen, the rhodopsin-phospho-Ser^334^ was higher in LL than in LD controls and was localized mainly in the OS of retina photoreceptors exposed to two days of LL stimulation. However at LL 4D and LL 7D, it was also found in the Pellet fraction. A quantitative analysis of the OS fraction showed a slight increase in rhodopsin-phospho-Ser^334^ between four to forty-eight hours of LL (Figure 5B); in contrast, at longer times of LL (LL 4D or LL 7D) the rhodopsin phosphor-Ser^334^ was higher and statistically significant after seven days of LL (Figure 5C). Moreover, the rhodopsin was completely dephosphorylated after seven days in DD (Figure 5A). These results suggest that higher levels of phosphorylation or delayed dephosphorylation activity occurred with constant low light stimulation.

## Discussion

Light-induced stress on the retina causes the apoptotic cell death of photoreceptors [29,45,46], with its severity depending on the light intensity, exposure duration and wavelength [10,11,47]. However, the molecular mechanisms giving rise to this response have not been fully elucidated to date.

In albino rats, retinal exposure to low white light leads to severe degenerative changes, with reduced thickness of the ONL and ultrastructural alterations such as fragmentation, disorientation of the OS and changes in the amplitude of the electroretinogram b-wave [13,26-29]. These phenomena, however, are not found in rats exposed to cyclic low-intensity light (less than 270 lx) lower than the threshold of cyclic light intensity capable of producing damage to the retina [32]. Our data show that constant light, but not cyclic exposure to 200 lx, produced photoreceptor death in albino Wistar rats (Figure 1). Nevertheless, we cannot rule out the possibility of ultrastructural alterations having occurred under both LL and LD exposure. Retinal ONL reduction was statistically significant after only seven days of low light exposure compared with animals kept in DD or at regular LD cycles of 200 lx intensity, indicating that cell damage processes may be slower than bright light damage and correspond to a different, phototransduction-dependent cell death mechanism. In this connection it is known that photoreceptor apoptosis induced by constant light exposure at lower intensities requires the activation of photopigments and consequent downstream signal transduction [25], suggesting that impairment of the phototransduction mechanism could be responsible for cell death.

Flow cytometry and TUNEL assays revealed cell death in retinas exposed to LL after four days, with retinal cell death and an increase in Annexin V being observed after five days of light stimulation with a concomitant increment in PI (Figure 2A,B). TUNEL analysis showed positive staining in the ONL area (see Figure 2B), demonstrating that all cell deaths were of photoreceptors.

Caspase-3 is recognized as being one of the key executioners of apoptosis. However, the apoptotic pathway and the role of caspase-3 in light-induced models depend on multiple factors such as animal strain, light intensity and the wavelength used. Some authors have attributed a central role to this enzyme in photoreceptor degeneration [18-20,48], while others have reported a caspase-3-independent mechanism involving Ca^+2^-dependent protease calpains or cathepsin D as alternative death pathways [20-24]. To try to elucidate the type of mechanisms involved in this phenomenon, we systematically studied caspase-3 protein expression and enzymatic activation applying antibodies against activated and non-activated isoforms using a commercial colorimetric activity assay kit. As shown in Figure 3, neither method was able to detect caspase-3 activity at any time of the light exposures studied. Although the apoptosis mechanism is known to be involved after retinal damage, other pathways such as necroptosis might also play a role. In this sense, Trichonas et al. [49] demonstrated that receptor-interacting protein (RIP) kinase-mediated necroptosis death was implicated in the retinal detachment induced by subretinal injection of sodium hyaluronate, indicating that RIP may be a complementary mechanism of photoreceptor cell death. Here, we show that photoreceptor cell death in retinas exposed to LL occurred through a caspase-3 independent mechanism, suggesting that photoreceptor cell death is an apoptotic caspase-3 independent mechanism. Nevertheless, the participation of a necroptosis mechanism cannot be ruled out. Further studies are necessary to determine whether Ca^+2^-dependent protease calpains or cathepsin D apoptotic processes, necroptosis or indeed both mechanisms are involved in photoreceptor cell death.

Quantitative analysis of rhodopsin expression revealed a significant reduction in the protein after seven days of LL, coinciding with a reduction in the number of photoreceptor cells (Figure 4) and thus suggesting that constant low light stimulation does not affect rhodopsin expression before photoreceptor cell death. However, western blot analysis revealed an increase in the positive immunoreactivity of rhodopsin-phospho-Ser^334^ at different times of LL exposure (Figure 5). Although these increments were not statistically significant at short times of LL (between LL 4 h to LL 48 h), at a longer time (LL 7D) the rhodopsin-phospho-Ser^334^ was higher and significant.

Depending on the mammal species, six or seven potential phosphorylation sites in the C-terminus of rhodopsin [50] Ser^334^ and Ser^338^ have been identified as major sites for the phosphorylation of rhodopsin in vivo [51], so as to obtain maximal high affinity binding of arrestin-1 [52]. Phosphorylation occurs in a sequential manner, correlating with the degree of illumination [53]. Thus, under high levels of illumination, rhodopsin is phosphorylated at relatively higher levels for the mono-, di-, tri-, and tetraphosphorylated species than for the penta- and hexaphosphorylated species [54], resulting in maximum arrestin binding and receptor desensitization [55].

Studies on the retinal photoreceptor degenerative model of cancer-associated retinopathy (CAR) have revealed significantly high levels of rhodopsin phosphorylation [56-58]. Rhodopsin mutants causing autosomal dominant retinitis pigmentosa are also phosphorylated at significantly higher levels than in wild-type cells [59], and rhodopsin dephosphorylation kinetics in the retinal degeneration of P23H rats have been shown to be extremely prolonged, especially in Ser^344^ [60]. Furthermore, mice with a missense mutation (K296E) that produces an opsin with a non-chromophore binding site (spontaneously activated) present phosphorylated rhodopsin tightly bound to arrestin in vivo [61]. In this sense, Alloway et al. [62] demonstrated that in certain genetic backgrounds in Drosophila, the light-dependent formation of stable phosphorylated rhodopsin-arrestin complexes, which mostly accumulate in the inner segment of photoreceptor cells, may constitute a molecular mechanism for the initiation of retinal degeneration.

It has been shown that the level and multiplicity of rhodopsin phosphorylation were reduced as a function of exposure duration in retinas of Sprague-Dawley (SD) rats subjected to light stress in the form of exposure to intense green light for up to eight hours [54]. These authors hypothesize that as regenerated rhodopsin is constantly re-activated by light, the supply of 11-cis retinal declines gradually and reduces the level of rhodopsin phosphorylation. However, SD and Royal College of Surgeons (RCS) rats exposed to white light for 24 h showed a significant delay in the kinetics of rhodopsin dephosphorylation in Rhodopsin Ser^334^ and Ser^338^, with high levels of phosphorylated rhodopsin being demonstrated by immunofluorescence labeling [57]. This discrepancy may be due to the rhodopsin phosphorylation residue studied or a result of the light treatment, with a lower intensity of green light or the full spectrum of white light perhaps being necessary to produce the effect. Taken together, all these results suggest that defects in the regulation of the phototransduction cascade by rhodopsin phosphorylation and the consequent internalization of the rhodopsin-arrestin complex may constitute a key step in photoreceptor degeneration by mutations in key components of phototransduction or light stress. Our studies suggest that rhodopsin phosphorylation may be affected by prolonged phototransduction mechanism activity and generates a rise in phosphorylation and/or delayed dephosphorylation of rhodopsin. Further studies will be required to investigate the phosphorylation state in other residues of rhodopsin and internalization to the inner segment in LL by low light and to examine whether alterations in RK and PKC activity are dependent on the duration of the light stimulus.

Based on our results discussed here, we conclude that constant illumination of low white light intensity (200 lx) produced retinal degeneration through a caspase-3-independent mechanism, whereby the ONL was significantly reduced after seven days of constant stimulation. One of the key mechanisms for the initiation of this process could be rhodopsin hyper-phosphorylation as a consequence of impairment of the enzymes related to the phosphorylation/dephosphorylation processes, and internalization to the photoreceptor cell soma. In this sense, constant low light-induced photoreceptor cell death could be a useful model for studying the mechanisms involved in phototransduction defects at the molecular level. As the most common form of retinal degeneration results from a primary defect in rods with secondary cone loss [5,63], the use of such a model in the comprehensive study of retinal degeneration may thus provide a basis for future therapies to prevent or at least delay visual cell loss.

## Appendix 1. Effect of constant low light exposure on central retinal thickness.

To access the data, click or select the words “Appendix 1.” **A-C:** Retinas reared in a 12:12 h light (200 lx): dark cycle (LD Cycle). **D-F**: Retinas exposed to constant low light for two days (LL 2D). **G-I**: Retinas exposed to constant low light for ten days (LL 10D), **A,D,G:** Retinas labeled with PI; **B,E,H:** Transmittance image. Scale bar indicates 30 µm **C,F,I:** Transmittance image. Scale bar indicates 15 µm. ONL, outer nuclear layer; INL, inner nuclear layer; GCL, ganglion cell layer; OS, outer segment; IS, inner segment.
